# Concurrent Hepatic Abscess and Pleural Empyema due to *Actinomyces odontolyticus* After Recent COVID‐19 Infection: A Case Report

**DOI:** 10.1155/crdi/8846561

**Published:** 2026-06-28

**Authors:** Dimo Dimitrov, Jad Mitri, Hind El Naamani, Claudia Nader

**Affiliations:** ^1^ Department of Medicine, Boston Medical Center-Brighton, Boston, Massachusetts, USA; ^2^ Boston University School of Medicine, Boston, Massachusetts, USA, bu.edu; ^3^ Division of Infectious Diseases, Boston Medical Center-Brighton, Boston, Massachusetts, USA

**Keywords:** *Actinomyces odontolyticus*, actinomycosis, case report, empyema, hepatic abscess, post-COVID-19 infection

## Abstract

**Introduction:**

Actinomycosis is a rare, slow‐progressing infection mimicking malignancies by penetrating mucosal barriers and spreading across anatomical planes. While typically affecting the cervicofacial area, it can also involve the thorax and abdomen, with simultaneous hepatic and pulmonary involvement being particularly rare.

**Case Description:**

We report the case of an 85‐year‐old woman with chronic kidney disease, diabetes, and multiple dental procedures who developed fever, myalgias, and abdominal pain a few weeks after recovering from COVID‐19. Diagnostic workup revealed a liver abscess extending into the right perinephric space and a small pleural effusion. Gram stain of abscess aspirate showed Gram‐positive, branching filamentous rods, negative on a modified AFB stain. Cultures grew *Actinomyces odontolyticus*, differing from the more common *A. israelii*. Her hospital course was complicated by acute hypoxic respiratory failure, with CT showing an expanded, loculated right pleural effusion. Treatment included prolonged antibiotic therapy and interventional radiology procedures for abscess drainage.

**Discussion:**

This case highlights the diagnostic and therapeutic challenges of hepatic actinomycosis caused by *A. odontolyticus*, particularly when affecting adjacent structures, such as the diaphragm and lung. The infection may have spread hematogenously to the liver with subsequent transdiaphragmatic extension into the thoracic cavity or, alternatively, may have originated from aspiration‐related thoracic infection with secondary extension toward the liver. Typically occurring in immunocompetent individuals, this severe presentation may reflect the combined influence of recent COVID‐19 infection, chronic comorbidities, and recent dental procedures. This underscores the need for vigilance in recognizing atypical postviral infections and highlights the need for further research into opportunistic bacterial infections following COVID‐19‐related immune dysregulation.

**Highlights**
- Rare case of hepatic and pulmonary actinomycosis post‐COVID‐19 in an elderly patient.
- A rare report of Actinomyces *odontolyticus* causing liver abscess with concurrent pleural empyema.
- Diagnostic challenges include distinguishing hematogenous vs. aspiration routes of infection.
- Successful management with prolonged antibiotics and multiple drainage procedures.
- Recent COVID‐19 infection may represent an additional host‐susceptibility factor in a multifactorial presentation.

## 1. Introduction

Actinomycosis poses a diagnostic challenge for clinicians owing to its diverse clinical presentations, infrequent occurrence, and indolent progression. This rare bacterial infection, caused by Actinomyces spp., most commonly *Actinomyces israelii*, is characterized by its ability to breach mucosal barriers, form fistulas, and sinus tracts, facilitating contiguous spread through anatomical structures and extending to the skin, adjacent organs, or even bone. In rare instances, hematogenous seeding may lead to infection at distant sites, further complicating diagnosis and management, often mimicking malignancies [1, 2].

Cervicofacial involvement is the most prevalent form of actinomycosis. Thoracic cases, comprising about 15% of total occurrences [3], affect the lungs in half of the cases, presenting as pleural thickening, effusion, or empyema. Abdominal infections account for 20% of the cases, with hepatic actinomycosis representing 15% of these—equating to around 5% of all cases [4, 5]. These infections typically present as single or multiple abscesses or masses [6].

## 2. Case Presentation

An 85‐year‐old woman with a history of CKD Stage 5, Type 2 diabetes, hypertension, and a COVID‐19 infection one month prior presented with a 2‐week history of fever, myalgias, and abdominal pain. On arrival, she was febrile to 101 F, hemodynamically stable, and maintaining an oxygen saturation above 95% on room air. Physical examination revealed tenderness in the left lower quadrant. Blood work demonstrated a white‐blood‐cell count of 18.3 × 10^3^/μL, hemoglobin of 8.1 g/dL, impaired renal function with creatinine of 3.4 mg/dL, and elevated transaminases AST/ALT 43/25 U/L, with normal total bilirubin 0.6 mg/dL and alkaline phosphatase 90 U/L. CT scan showed a collection in the right liver lobe extending into the posterior perihepatic space and further into the perinephric space (Figure 1). Two sets of blood cultures were sent but remained sterile.

**FIGURE 1:**
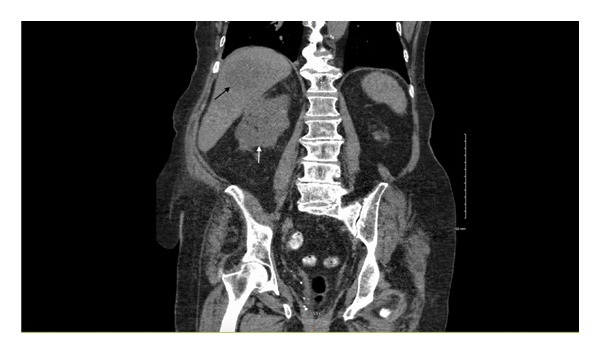
CT abdomen/pelvis without contrast demonstrating intrahepatic (black arrow) and perirenal abscesses (white arrow), frontal view.

Empiric antibiotic therapy with ceftriaxone and metronidazole was initiated. Two IR‐guided drains were placed: one intrahepatically in the right lobe and another in the perinephric space, both yielding purulent fluid. Gram stain from both specimens showed Gram‐positive filamentous rods with branching appearance (Figure 2). To differentiate from Nocardia, a modified acid‐fast staining was used and was negative, raising suspicion for Actinomyces as the culprit organism. Based on these results, the antibiotic regimen was changed to ampicillin/sulbactam. On the sixth day of incubation, bacterial colonies appeared on the anaerobic plates (Figure 3). Using the VITEK MS MALDI‐TOF (bioMérieux), the organism was subsequently identified as *Actinomyces odontolyticus*, leading to the definitive diagnosis.

**FIGURE 2:**
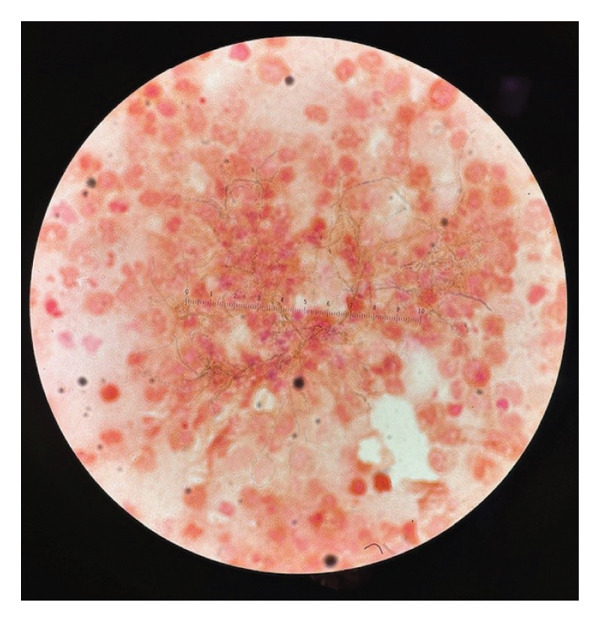
Filamentous rods with branching appearance observed through a microscope, highlighted using Gram‐staining technique.

**FIGURE 3:**
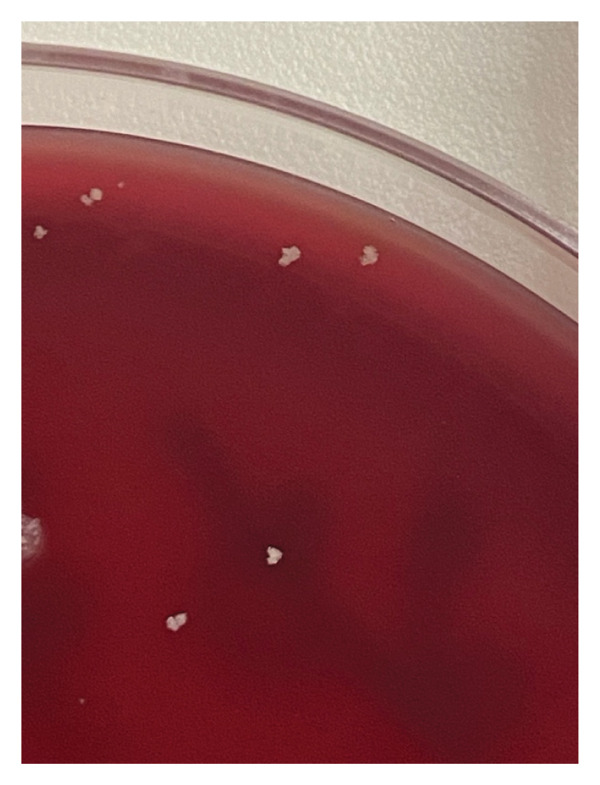
Actinomyces colonies growing on the anaerobic plate.

Despite initial drainage, abscess management remained challenging. The right lobe intrahepatic drain was displaced, and subsequent attempts to aspirate pus were unsuccessful, leading to interventional radiology (IR) to defer the placement of another intrahepatic drain. During the same procedure, aspiration of a newly discovered posterior perihepatic collection yielded 10 mL of fluid without the need for drain placement as ultrasound revealed complete resolution. Sixty mL of fluid was aspirated from the perinephric collection, revealing persistent Actinomyces infection. Interval CT scans showed a reduction in the perinephric fluid collection but no change in the intrahepatic collection, necessitating additional intrahepatic drainage while the perinephric drain remained.

During the hospital stay, the patient developed dyspnea, and imaging revealed an increased right‐sided loculated pleural effusion. A chest tube was inserted, and intrapleural tPA and DNase were instilled. After 9 days of drainage, the initial chest tube was replaced with a new one in a different empyema pocket. Further attempts to administer intrapleural tPA and DNase were halted due to a drop in hemoglobin and severe chest pain reported by the patient. Follow‐up chest CT showed a slight reduction in the pleural effusion.

Due to her refractory loculated pleural effusion, she was considered but deemed unsuitable for video‐assisted thoracoscopic surgery. The patient received a peripherally inserted central catheter (PICC) line and was discharged on a prolonged antibiotic regimen with ampicillin/sulbactam 3 g every 24 h, with a plan to complete 6 weeks of intravenous therapy followed by 3–6 months of oral antibiotics. Additionally, nutritional support, including supplements and dronabinol for appetite stimulation, was provided due to the patient’s malnourished status. She subsequently followed up at an outside institution and did not follow up with our team. Based on available records, she clinically recovered. A detailed clinical timeline is provided in Table 1.

**TABLE 1:** Clinical timeline.

Time point	Clinical event
9 months before admission	Dental crown placement.

6 months before admission	Dental implant placement.

∼1 month before admission	COVID‐19 infection; recovered without hospitalization or targeted treatment.

2 weeks before admission	Developed fevers, myalgias, anorexia, abdominal discomfort/nausea, and weight loss

Hospital Day 0 (1/19/2024)	Presented/admitted with fever, leukocytosis, and abdominal pain. CT showed a right hepatic abscess with extension into the posterior perihepatic space and right perinephric space. Empiric treatment with ceftriaxone and metronidazole.

Hospital Day 1 (1/20/2024)	IR placed 2 drains: one in the right intrahepatic collection and one in the perinephric collection; both yielded purulent fluid.

Hospital Day 3 (1/22/2024)	PICC line placed in anticipation of long‐term IV antibiotics. Patient developed atrial fibrillation with RVR, managed with beta‐blocker titration. CXR showed a new right‐sided loculated pleural effusion/empyema; chest tube was inserted.

Hospital Days 4–13 (1/23‐2/1/2024)	Treated with chest tube drainage and intrapleural tPA/DNase, completing 6 instillations. The first chest tube was removed on 2/1 after that pocket was drained.

Hospital Day 6 (1/25/2024)	Gram stain showed Gram‐positive branching filamentous rods; modified AFB stain was negative, raising suspicion for Actinomyces rather than Nocardia. Antibiotics changed to ampicillin/sulbactam.

Hospital Day 13 (2/1/2024)	Right intrahepatic drain was displaced, thus removed. IR attempted aspiration in the drain region but could not aspirate pus, so no replacement intrahepatic drain was placed. A new chest tube was placed in a different empyema pocket.

Hospital Day 16 (2/4/2024)	IR aspirated a right liver lobe perihepatic/subcapsular complex collection, yielding ∼10 mL fluid; Gram stain again showed gram‐positive rods concerning for persistent Actinomyces. Additional drainage was performed around the perihepatic/perinephric collection, but no additional drain was placed.

Hospital Day 18 (2/6/2024)	Repeat CT chest/abdomen/pelvis showed slight decrease in the loculated right pleural effusion and perinephric collection, but no change in the right intrahepatic collection. Additional tPA/DNase was attempted but stopped after severe chest pain, bloody/clotted output, and hemoglobin drop.

Hospital Day 19 (2/7/2024)	Continued on renally dosed ampicillin/sulbactam. Further lytic therapy was held due to bleeding/anemia.

Hospital Day 20 (2/8/2024)	Persistent thick Actinomyces‐associated collections with limited drain output; aggressive flushing of chest tube and perinephric drain continued. General surgery and thoracic surgery did not recommend operative intervention.

Hospital Day 21 (2/9/2024)	Chest tube output remained low despite flushing. Pulmonology noted limited success with drainage and suggested considering chest tube removal and long‐term antibiotic therapy; patient was not considered a surgical candidate.

Hospital Day 22 (2/10/2024)	Hemoglobin dropped to 6.9 g/dL; transfused 1 unit PRBC. Chest tube and perinephric drain had minimal output; transfer to outside hospital for further surgical/source‐control evaluation was discussed.

Hospital Day 23 (2/11/2024)	Patient hemodynamically stable, on room air, without fever/chills/chest pain. Chest tube and perinephric drain continued to have minimal output.

Hospital Day 24 (2/12/2024)	Patient discharged on renally dosed IV ampicillin/sulbactam 3 g every 24 h, with plan for 6 weeks IV therapy followed by oral antibiotics for 3–6 months.

## 3. Discussion

This case presents a unique occurrence of actinomycosis concomitantly affecting the liver and right pleural space. Furthermore, the identified pathogen, *A. odontolyticus,* contrasts with the more frequently encountered *Actinomyces israelii* [7, 8].

*A. odontolyticus* is part of the normal oral flora and resides on tooth surfaces and dental plaque [2]. It is the predominant subspecies in biofilm formation on tooth surfaces across all ages, interestingly independent of periodontal disease status [2]. Instances of this organism isolated from the lungs and liver have been described separately [2, 7] but not simultaneously. For example, Chao et al. [7] reported a liver abscess caused by *A. odontolyticus* a month after an artificial tooth replacement. Similarly, our patient received a dental implant and a crown at six and nine months, respectively, before presenting with involvement of both the liver and lungs, raising questions about the origin of the infection.

The route of dissemination in this case cannot be determined with certainty. One possible scenario could have been a hematogenous spread to the liver with subsequent extension to the thoracic cavity by contiguous spread, despite the negative blood cultures on admission. This hypothesis is supported by the initial findings of a liver abscess with perihepatic and perirenal involvement, accompanied by initially a small right pleural effusion without significant respiratory symptoms. Alternatively, the infection could have started in the lungs due to aspiration of the organism and subsequently spread to the liver. Although the initial chest x‐ray was clear and CT abdomen/pelvis showed only mild pleural effusion, the limitations of these studies might have underestimated the initial degree of lung involvement in the absence of a dedicated chest CT scan at presentation. The abrupt increase in oxygen requirements of our patient prompted the acquisition of a chest CT, which revealed extensive right‐sided pleural disease. This sudden onset of shortness of breath was unusual for Actinomyces and may have been caused by concurrent atelectasis.

As previously mentioned, hepatic involvement by itself is rare, affecting 5% of the cases of actinomycosis. Between 2000 and 2020, a total of 64 cases of hepatic actinomycosis have been identified, as outlined by the systematic review of Chegini et al. [8]. Most of the cases described were males (64%) and immunocompetent. Sharma et al. [9], who reviewed English literature, found 56 cases of hepatic actinomycosis, had similar findings—70.2% were male, mostly immunocompetent. In contrast, our patient was female with several chronic conditions that can potentially reduce immunity, including CKD Stage 5, obesity, and Type 2 diabetes mellitus [10, 11].

Interestingly, our patient’s symptoms started a month after recovering from COVID‐19 infection, which had not required hospitalization or targeted therapy. To our knowledge, no previous cases of hepatic actinomycosis with pleural empyema have been reported in the setting of recent COVID‐19 infection. Lévay et al. [12] described a case of actinomycotic liver abscess following COVID‐19 infection. Notably, they discuss the potential effects of SARS‐CoV‐2 on vascular permeability by binding to the ACE2 receptor, reducing its activity, and triggering the kallikrein–bradykinin pathway. In our patient, recent COVID‐19 infection may have contributed to host susceptibility alongside more established risk factors, including advanced age, diabetes, chronic kidney disease, and recent dental procedures. The literature contains only a few other cases involving Actinomyces and COVID‐19, describing actinomycotic sinomaxillary infections in a COVID‐19 patient [12–16]. Further research in this area is needed to provide answers to some of the outstanding questions.

Our patient received a prolonged course of renally dosed ampicillin/sulbactam, which is well‐known to be effective in treating actinomycosis. Other effective treatment modalities include puncture drainage and/or surgical resection. In this case, the patient received multiple percutaneous liver and pleural drainage procedures for source control, consistent with previous reports. Her successful discharge demonstrates that, despite its atypical presentation and challenging diagnosis and treatment, most cases of actinomycosis have positive outcomes [8, 9].

## 4. Conclusion

This case describes an unusual presentation of *A. odontolyticus* infection involving hepatic abscess and concurrent right pleural empyema in an elderly woman with recent dental procedures, chronic comorbidities, and recent COVID‐19 infection. It highlights the diagnostic challenge of actinomycosis, the importance of microbiologic confirmation and source control, and the need to consider atypical dissemination when hepatic and thoracic collections coexist. Patient‐ and disease‐specific factors, including an uncommon Actinomyces species, impaired host immunity, and recent COVID‐19 infection, may contribute to atypical presentations and warrant further research.

## Author Contributions

Dimo Dimitrov performed clinical data collection, drafted the initial manuscript, and contributed to the literature review and manuscript revision. Jad Mitri and Hind El Naamani contributed to clinical data collection, literature review, and manuscript revision. Claudia Nader supervised the case interpretation, infectious diseases management discussion, and critically revised the manuscript. All authors reviewed the final manuscript.

## Funding

This research did not receive any specific grant from funding agencies in the public, commercial, or not‐for‐profit sectors.

## Disclosure

This case was previously presented as a poster at the American College of Gastroenterology (ACG) Annual Scientific Meeting, held on October 28, 2024, in Philadelphia, PA, USA. All authors have approved the final manuscript.

## Ethics Statement

Institutional review board approval was not required for this single‐patient case report in accordance with institutional policy.

## Consent

Written informed consent was obtained from the patient for publication of this case report and any accompanying images.

## Conflicts of Interest

The authors declare no conflicts of interest.

## Data Availability

The data supporting the findings of this study are available from the corresponding author upon reasonable request.
